# Elastin homeostasis is altered with pelvic organ prolapse in cultures of vaginal cells from a lysyl oxidase‐like 1 knockout mouse model

**DOI:** 10.14814/phy2.14436

**Published:** 2020-06-12

**Authors:** Slater A. Jameson, Ganesh Swaminathan, Shataakshi Dahal, Bruna Couri, Mei Kuang, Anna Rietsch, Robert S. Butler, Anand Ramamurthi, Margot S. Damaser

**Affiliations:** ^1^ Department of Biomedical Engineering Lerner Research Institute Cleveland Clinic Cleveland OH USA; ^2^ Department of Bioengineering Rice University Houston TX USA; ^3^ Department of Quantitative Health Sciences Cleveland Clinic Lerner Research Institute Cleveland OH USA; ^4^ Department of Molecular Medicine Cleveland Clinic Lerner College of Medicine of Case Western Reserve University Cleveland OH USA; ^5^ Advanced Platform Technology Center Louis Stokes Cleveland Department of Veterans Affairs Medical Center Cleveland OH USA

**Keywords:** cross‐link, extracellular matrix, matrix metalloproteinase, tissue inhibitors of matrix metalloproteinase, tropoelastin

## Abstract

Pelvic organ prolapse (POP) decreases quality of life for many women, but its pathophysiology is poorly understood. We have previously shown that Lysyl oxidase‐like 1 knockout (*Loxl1* KO) mice reliably prolapse with age and increased parity, similar to women. Both this model and clinical studies also indicate that altered elastin metabolism in pelvic floor tissues plays a role in POP manifestation, although it is unknown if this is a cause or effect. Using *Loxl1* KO mice, we investigated the effects of genetic absence of *Loxl1*, vaginal parity, and presence of POP on the expression of genes and proteins key to the production and regulation of elastic matrix. Cultured cells isolated from vaginal explants of mice were assayed with Fastin for elastic matrix, as well as RT‐PCR and Western blot for expression of genes and proteins important for elastin homeostasis. Elastin synthesis significantly decreased with absence of LOXL1 and increased with parity (*p* < .001), but not with POP. Cells from prolapsed mice expressed significantly decreased MMP‐2 (*p* < .05) and increased TIMP‐4 (*p* < .05). The results suggest changes to elastin structure rather than amounts in prolapsed mice as well as poor postpartum elastin turnover, resulting in accumulation of damaged elastic fibers leading to abnormal tropoelastin deposition. POP may thus, be the result of an inability to initiate the molecular mechanisms necessary to clear and replace damaged elastic matrix in pelvic floor tissues after vaginal birth.

## Graphical abstract

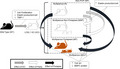

## 1 INTRODUCTION

Pelvic organ prolapse (POP) is characterized by the downward descent and abnormal protrusion of pelvic organs, which reduces quality of life for many women (Jelovsek et al., 2007). Current approaches to manage POP have high complication and revision rates (Eilber et al., 2013; Vandendriessche et al., 2017). As such, the Food and Drug Administration (FDA) has warned against the use of vaginal meshes for POP (Mucowski et al., 2010) which, in some cases, have resulted in their removal from the market.

Risk factors for POP include vaginal delivery, advanced age, hysterectomy, and abnormalities of connective tissue (Jelovsek et al., 2007). The extracellular matrix (ECM) of pelvic floor tissues plays a critical role in imparting structural integrity to pelvic tissues and undergoes extensive remodeling during pregnancy and after childbirth, including increased synthesis of elastin and its assembly into elastic fibers (Heng et al., 2012; Ulrich et al., 2014; Word et al., 2007). Although the mechanism of POP initiation is not known, studies in both animal models and human subjects have demonstrated abnormal structural ECM homeostasis in prolapsed tissues (Budatha et al., 2011; Zhou et al., 2012). Evidence of altered elastic matrix homeostasis in animal models of POP suggests that defective postpartum remodeling of elastic fibers, which provide tissue stretch and recoil properties, contribute to POP development (Drewes et al., 2007; Liu et al., 2006). We have corroborated these findings in lysyl oxidase‐like‐1 (*Loxl1*) knockout (KO) mice, which reliably prolapse with increased age and parity, similar to outcomes in women (Couri et al., 2012). Evidence of significantly reduced LOXL1 expression in tissues from POP patients further justifies the relevance of our mouse model to the study of POP (Alarab et al., 2010; Klutke et al., 2008; Zhao & Zhou, 2012). These similarities to the human condition suggest that *Loxl1* KO mice are appropriate to investigate changes in elastic fiber homeostasis and its relation to how POP develops after vaginal delivery.

Besides LOXL1, numerous other proteins regulate elastic matrix synthesis and repair. Elastic fiber neoassembly begins with extracellular secretion of tropoelastin monomers that self‐assemble (coacervate) into insoluble aggregates (Kielty et al., 2002; Wagenseil & Mecham, 2007), a process mediated by the LOX protein family, which cross‐link the lysine residues of tropoelastin, and by fibulin‐5 which prevents excess tropoelastin aggregation (Papke & Yanagisawa, 2014; Wagenseil & Mecham, 2007). These aggregates are then deposited onto microfibrillar scaffolds rich in fibrillin‐1 (Wagenseil & Mecham, 2007). Fibulin‐5 facilitates the colocalization of the scaffold proteins, tropoelastin aggregates, and LOX family proteins on the cell surface (Papke & Yanagisawa, 2014; Yanagisawa et al., 2002). These coacervates undergo further cross‐linking and extend to form mature elastic fibers (Kielty et al., 2002).

The elastic matrix is maintained by the tight regulation of elastic fiber metabolism by matrix metalloproteinase (MMP) enzymes, and their antagonists, tissue inhibitors of metalloproteinases (TIMPs) (Clark et al., 2008; Wieslander et al., 2008). Furthermore, factors such as transforming growth factor beta (TGF‐β) and bone morphogenetic protein 1 (BMP‐1) also indirectly promote elastic matrix deposition via interaction with LOX and LOXL1 respectively (Borel et al., 2001; Shanley et al., 1997). The disruption of normal elastic fiber homeostasis has been implicated in clinical manifestation of POP (Alarab et al., 2010; Chen & Yeh, 2011; Chen et al., 2002; Jackson et al., 1996; Qi et al., 2011; Shynlova et al., 2013). However, there has been no controlled study to ascertain the individual contributions of parity, prolapse, and LOXL1 absence on maintenance of elastic fiber homeostasis. In this study, we used the *Loxl1* KO mouse model to investigate changes to elastic fiber neoassembly with vaginal delivery, POP, and the absence of LOXL1.

## 2 MATERIALS AND METHODS

2

### 2.1 Animal breeding techniques and tissue harvest

Research involving animals was performed with approval from the Cleveland Clinic Institutional Animal Care and Use Committee. *Loxl1* KO female mice were housed with males as single pairs and allowed to breed ad libitum until attaining multiparity at 30 weeks of age. Six *Loxl1* KO multiparous nonprolapsed (MNP) and five prolapsed (MP) mice were used. Three nulliparous (N) *Loxl1* KO and six virgin wild type (WT) mice (Jackson Labs hybrid C57B1/6 and Sv129) were used as controls.

Whole vaginal tissue was harvested from the mice under isoflurane anesthesia via a midline abdominal incision. The bladder and urethra were dissected from the anterior vaginal wall. The vagina was dissected from the rectum and transected at the level of the cervix proximally and at the skin distally. Vaginal tissues were placed in Dubecco's Modified Eagle's Medium (DMEM)/F12 medium (Invitrogen, Carlsbad) with 20% v/v fetal bovine serum (FBS; Invitrogen) and 1% v/v penicillin‐streptomycin (Penstrep; ThermoFisher, South Logan) for tissue digestion and cell isolation.

### 2.2 Cell isolation and culture

Primary nonepithelial vaginal cells (NEVCs) were isolated from harvested vaginal tissues by enzymatic digestion as published (Eilber et al., 2013; Ekman‐Ordeberg & Dubicke, 2012). Briefly, the harvested tissues were cut into small pieces (5 × 5 mm), digested in DMEM/F12 medium containing 20% v/v FBS, 1% v/v Penstrep, and 125 U/mg collagenase (Worthington Biochemicals, Lakewood) for 20 min at 37°C, centrifuged at (50*g*, 1 min), and the supernatant was aspirated. The tissues were again digested (1 hr, 37°C) with the digestion solution also containing 15 U/mg of elastase (Sigma‐Aldrich; St. Louis). The digestate was centrifuged (400*g*, 5 min) the cell pellet resuspended in DMEM/F12 medium containing 20% v/v FBS, and 1% v/v Penstrep and seeded in 6‐well plates at 3 × 10^4^ cells/well. Attaching primary NEVCs were propagated in DMEM/F12 containing 10% v/v FBS and passaged at confluence. Nonprimary cells (<P6) were then seeded for experiments in six‐well plates at 50,000 cells/10 cm^2^ (*n* = 6 replicate cultures per animal). Since we have previously shown that 21 days of culture allows for reliable quantification of elastin by Fastin assay and other associated proteins by Western blot (Ramamurthi et al., 2012), we cultured the NEVCs for 21 days for western blot and Fastin^®^ assays. In 3‐week cultures, the high level of confluence and cell–cell contact inhibition, and abundant matrix presence can alter the native gene expression profile of SMCs (Liu, 2012). To avoid this and assess gene expression patterns reflective of differences in phenotype between our different groups of cells uninfluenced by cell crowding, contact inhibition, and matrix, NEVCs were cultured for 14 days before harvesting for gene expression by PCR. At this time point cell layers are subconfluent and matrix deposition is limited. Cells were also cultured for one day (time controls) to assess cell number increases over the culture period.

We have previously shown that these vaginal cells from *Loxl1* KO mice with POP express classic smooth muscle cell (SMC) markers, such as such as α‐smooth muscle actin, caldesmon, and tropomyosin (Ramamurthi et al., 2012). Additional characterization of NEVCs was done as part of this study using immunofluorescent staining for myosin heavy chain 11 (Abcam #ab683 mouse monofilament, 1:250), fibroblast‐specific protein‐1 (FSP‐1; Fisher Scientific #07‐227‐4MI Rabbit polyclonal, 1:100), and pan‐cytokeratin antibodies (AE‐1 & AE‐3: Abcam #ab80826, mouse monoclonal, 1:200) that are expressed by epithelial cells. Positive control cells for AE1 & AE3 were MLE12 (ATCC^®^ CRL‐2110), mouse lung epithelial cells. Secondary antibodies were donkey anti‐mouse IgG (H + L) #A‐21203, 1:1,000 (ThermoFisher Sci) and donkey anti‐rabbit IgG (H + L), #A‐21207, 1:1,000 (ThermoFisher Sci).

### 2.3 qRT‐ PCR

Total RNA was isolated from NEVCs seeded from wild type (WT), nulliparous *Loxl1* KO (N), and multiparous *Loxl1* KO mice that had either developed POP (MP) or did not (MNP) after 14 days in culture using an RNeasy mini kit (Qiagen, Valencia). Briefly, the NEVC layers were harvested in RNEasy Lysis Buffer buffer containing 1% w/v beta‐mercaptoethanol and stored in −80°C. RNA concentration was determined using a NanoDrop^®^ ND‐1000 Spectrophotometer (Thermo Scientific). Samples were reverse transcribed using SuperScript First‐Strand Synthesis kit (RT‐PCR, Invitrogen). The primers (Applied Biosystems, Grand Island) used were elastin (*Eln*, Mm00514670_m1), collagen 1a (*Col1a*, Mm00801666_g1), metalloproteinase (*Mmp*) *2* (Mm00439498_m1) and *9* (Mm00442991_m1), tissue inhibitor of metalloproteinases (*Timp*) *3* (Mm00441826_m1) and *4* (Mm01184417_m1), fibulin‐5 (*Fbln5*, Mm00488601_m1), fibrillin‐1 (*Fbn1*, Mm00514908_m1), lysyl oxidase (*Lox*, Mm00495386_m1), transforming growth factor beta‐1 (*Tgfb1*, Mm01178820_m1), and bone morphogenetic protein ‐1 (*Bmp1*, Mm00802220_m1). Reactions were performed using a TaqMan^®^ Real‐Time PCR master mix (Applied Biosystems) in an Applied Biosystem 7500 Detection System. The gene *18S* (4319413E) was used as an endogenous control and the standard curve method was utilized to determine the relative expressions of the target genes.

### 2.4 Western blots for matrix homeostasis proteins

COL1A, TIMP‐1, TIMP‐4, MMP‐2, MMP‐9, LOX, TGF‐β1, and BMP‐1 protein expression in cells seeded from WT mice and MNP, MP, and N *Loxl1* KO mice after 21 days in culture were assessed using western blot. Following 21 days in culture, NEVCs were harvested in Radioimmunoprecipitation buffer (Thermo Scientific) containing Halt™ protease inhibitor cocktail (Thermo Scientific), and three wells were pooled per replicate (*n* = 3 replicates/animal). Samples (20 µl/lane) were loaded under reduced conditions into a 10% sodium dodecylsulfate polyacrylamide gel electrophoresis gel (SDS‐PAGE) for analysis of MMP‐2 and −9, COL1A, and BMP‐1 proteins or a 12% SDS‐PAGE gel for LOX, TIMP‐1, TIMP‐4, and TGF‐β1 proteins. A BenchMark™ prestained molecular weight ladder (Invitrogen) and appropriate protein standards (positive controls) were also loaded onto the gels. The gels were wet transferred onto nitrocellulose membranes (Invitrogen), blocked for 1 hr with Odyssey Blocking Buffer (LI‐COR Biosciences, Lincoln, NE), and immunolabeled with primary antibodies (1 hr, 25°C), then secondary antibodies (1 hr, 25°C) and proteins then detected using a LI‐COR Odyssey scanning system. Table 1 shows a list of commercial antibodies used. Fluorescence intensities of the protein bands were quantified using Image Studio Lite software^®^ (LI‐COR Biosciences) and normalized to the intensities of their respective β‐actin bands (loading control).

**TABLE 1:** List of primary and secondary antibodies used for western analysis

Antibody	Primary/Secondary/Mol Wt	Mono/Poly	Dilution	Source	Species reactivity	Company/Catalog #
TIMP4	P 26 KD	P	1:2000	Rabbit	H, M, Rat	Abcam; Ab58425
TGFβ1	P 28 KD	P	1:1,000	Rabbit	H, M, Rat	Millipore; MAB0132
MMP9	P 92 KD	M	1:5,000	Rabbit	H, Rat	Millipore 04‐1150
β actin	P 43 KD	M	1:5,000	Mouse	H, M, Rat	Santa Cruz; Sc‐47778
TMP1	P 28 KD	P	1:1,000	Rabbit	H, M, Rat	LS‐C176561
MMP2	P 72 KD	P	1:2000	Rabbit	M, R, H, Chicken	Abcam; Ab3710
LOX	P 50 KD	M	1:1,000	Mouse	M, R, H	Santa Cruz; SC‐66948
BMP‐1	P 111 KD	P	1:1,000	Rabbit	M, H	Abcam; Ab38953
Col1a	P 70–90 KD	P	1:200	Goat	M, R, H	Santa Cruz; SC‐8784
Donkey anti‐mouse	S			Donkey		Li‐cor; 926–68072
Donkey ant‐rabbit	S			Donkey		Li‐cor; 926‐68072
Donkey anti‐goat	S			Donkey		Li‐cor; 926‐68072

### 2.5 DNA assay for cell proliferation

Cell counts in NEVC cultures were estimated from their DNA content. DNA amounts were measured in NEVC cultures at one day (time controls) and 21 days using the fluorometric assay of Labarca and Paigen (1980). Briefly, the cell layers (*n = *6 replicate cultures/animal) were scraped, harvested in NaCl/Pi buffer, sonicated and DNA amounts quantified using Hoechst 33258 dye (Sigma Aldrich). Cell number was calculated assuming 6 pg of DNA/cell (Labarca & Paigen, 1980).

### 2.6 Fastin assay for matrix elastin

Total matrix elastin deposited by the cell layers over 21 days was quantified using the Fastin^®^ assay, as published (Swaminathan, Sivaraman, et al., 2016, Swaminathan, Stoilov, et al., 2016, Swaminathan, Gadepalli, et al., 2017). Briefly, matrix elastin was isolated from cell layers collected in the previous step (Section 2.5) by double acid digestion. The samples were centrifuged (10,000*g*, 10 min), and the pellets incubated with 400 µl of 1 M oxalic acid (90°C, 1 hr), centrifuged (10,000 g, 10 min), and the supernatant collected. The undigested pellet was digested again (100 µl of 0.25 M oxalic acid; 90°C, 1 hr) and centrifuged (10,000*g,* 10 min). The supernatant was collected, mixed with the supernatant from first step, and matrix elastin amounts quantified using a Fastin^®^ assay (Accurate Chemical and Scientific Corporation, Westbury). Elastin content was normalized to the cell count (or DNA content) of the respective cell layers.

### 2.7 Statistical analysis

Results are presented as box plots or mean ± standard error of *n* = 5 WT, 3 N, 5 MNP, and 4 MP mice for PCR and western blot assays and *n* = 6 WT, 3 N, 6 MNP, and 5 MP for the Fastin and DNA assays. To assess MMP/TIMP balance, we also determined the following protein expression ratios: MMP‐2/TIMP‐1, MMP‐2/TIMP‐4, MMP‐9/TIMP‐1, and MMP‐9/TIMP‐4.

Data analysis assessed effects of LOXL1 absence, delivery, and of prolapse on elastin homeostasis proteins. Data were accordingly grouped into seven separate data sets: WT, N, MP, MNP, all multiparous mice (MULT: consisting of MP and MNP), all *Loxl1* KO mice (KO: consisting of N, MP, and MNP), and all *Loxl1* KO nonprolapsed mice (NON‐POP: consisting of N and MNP). To determine the effect of *Loxl1* KO on gene and protein expression, we compared WT versus KO and WT versus N outcomes. To elucidate effect of parity, we compared N versus MULT, and N versus MNP outcomes, and to determine the effect of prolapse, we compared outcomes in MP versus NON‐POP, MP versus N, and MP versus MNP cultures. Repeated measures mixed regression methods were used to compare gene and protein expression results as well as elastin content. Pairwise comparisons were adjusted for multiple comparisons using Bonferroni corrections since only certain pairwise comparisons were of interest. Data for some of the mouse groups for various qPCR measures exhibited a greater degree of variation when compared to other groups but this variation was not due to the existence of a few extreme points. After running the regression, the analysis of the residuals indicated that their distributions were acceptable with respect to use for assessing significant differences between mouse group means. Therefore, we did not exclude outliers from the data analysis. Significant differences between groups are reported for adjusted *p* < .05.

We also calculated regressions of the log–log comparisons of expression between pairs of different genes and proteins and the line fit slopes were tested for statistical significance. The gene–gene and protein–protein correlation line fit slopes deemed significant were then compared against one another to ascertain statistical significance of their differences, determined as *p* < .05. The significant or correlated slopes and their associated statistics resulting from the repeated measures regression of log‐transformed data were tested for significant differences between one another using methods outlined by Larson (Larson, 1992). To test specific hypotheses regarding differences due to the absence of LOXL1, delivery, and prolapse, only select comparisons of these individually significant slopes were tested for significance (*p* < .05). These comparisons were made only if the slopes of both groups in the contrast statement were deemed to be independently statistically significant or correlated. Thus, the number of contrasts tested for a given protein/gene combination varied. No corrections were made for multiple comparisons of significant slopes within a given protein or gene combination.

## 3 RESULTS

### 3.1 Cell types in NEVCs

Immunofluorescence studies to characterize the cell types contained in NEVCs demonstrated that NEVCs consist of SMCs and fibroblasts but not epithelial cells (Figure S1; https://doi.org/10.6084/m9.figshare.11798871), supporting the nomenclature of nonepithelial vaginal cells (NEVCs).

### 3.2 Effect of Loxl1 KO

**FIGURE 1:**
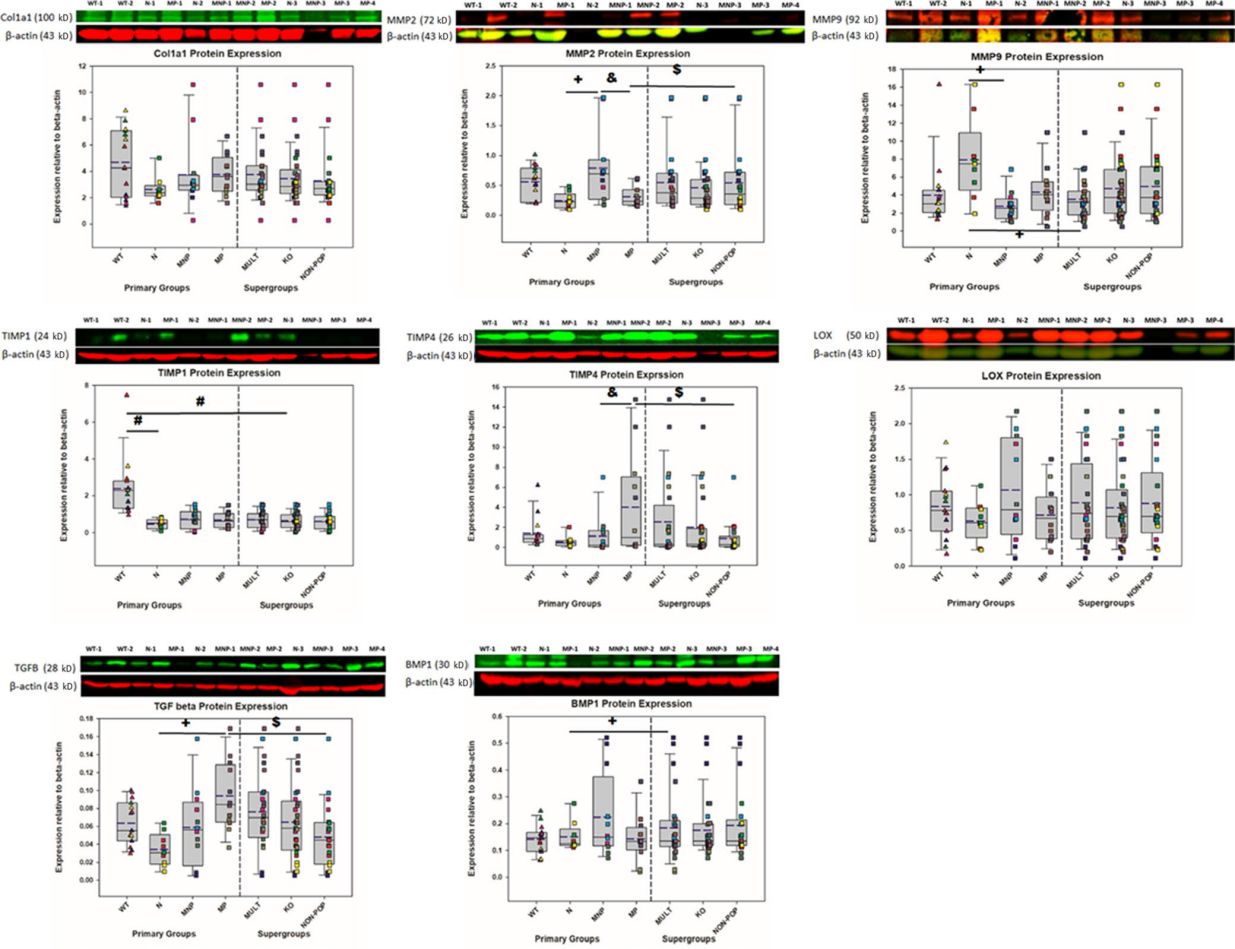
Western Blot results. Each bar in the box plot represents the mean with 25/75% confidence interval, the blue dotted lines indicate the mean. Data were collected from 3–5 nonepithelial vaginal cells (NEVCs) in each group. Each cell line has three replicates, as indicated above the images of the blots showing the bands for the protein of interest and the housekeeping protein (β‐actin; 43 kDa). NEVCs from wildtype (WT) mice are indicated as triangles to distinguish them from NEVCs in the other groups (squares) all of which are from lysyl oxidase‐like 1 knockout mice. The same color in the symbols correspond to samples from the same cell line. Repeated measures mixed regression methods with pairwise comparisons corrected using a Bonferroni correction. The following symbols denote a significant difference (*p* < .05) compared to the group in parentheses: # (WT), + (nulliparous; N), & (multiparous nonprolapsed; MNP), $ (multiparous prolapsed; MP). To be noted, the bands for the proteins of interests and the β‐actin appear either red or green as indicated, based on the fluorophore‐conjugated secondary antibody used for their detection

**FIGURE 2:**
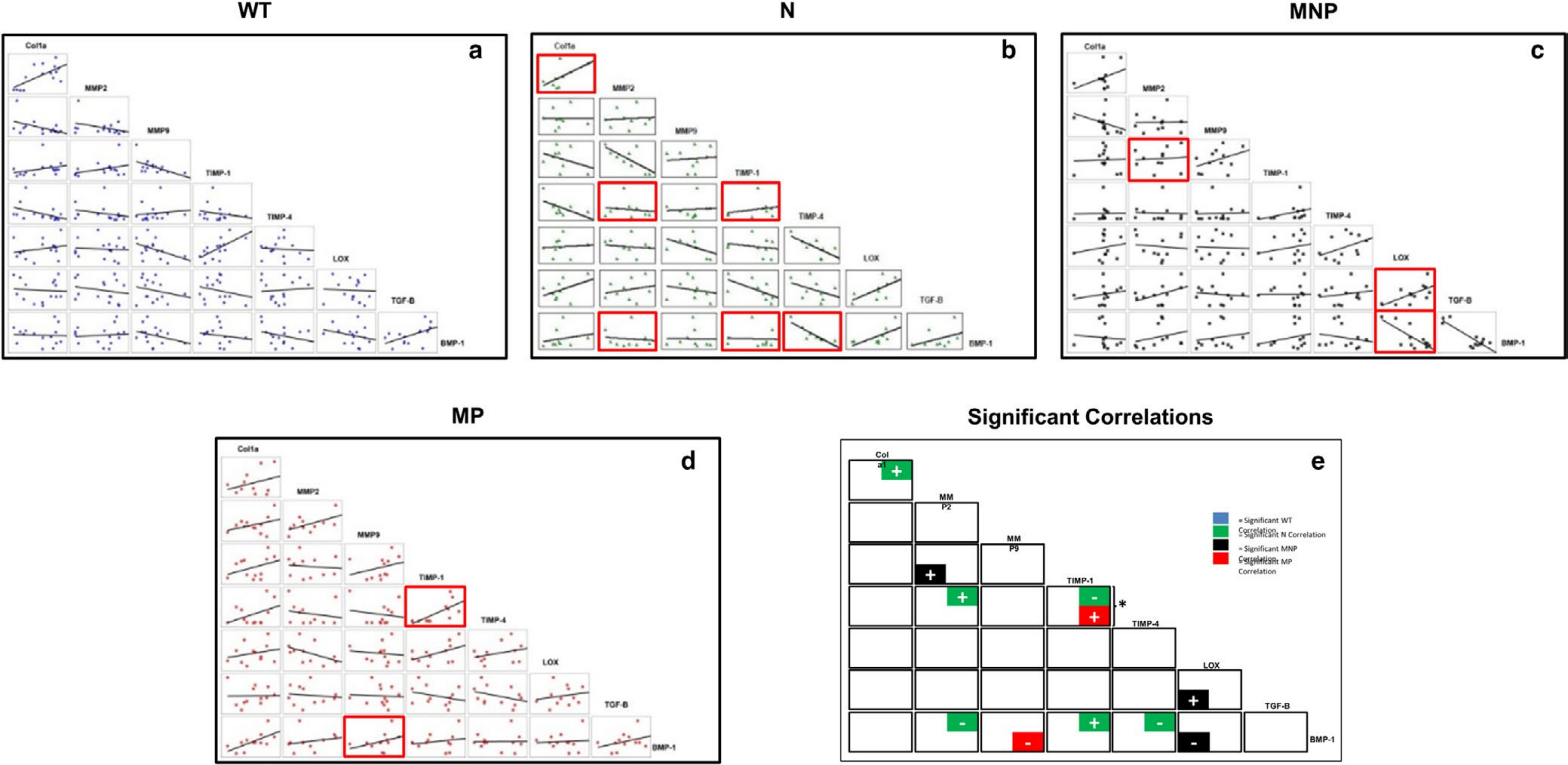
Protein‐pair correlation data from western blot results. Panels a–d show correlation data for wildtype (WT), nulliparous (N), multiparous nonprolapsed (MNP) and multiparous prolapsed (MP) mice with lines of best fit for each possible combination of proteins studied. Red boxes indicate significant correlation in expression amount between protein pairs (*p* < .05), or a statistically significant line fit. +/− indicate positive and negative correlations between protein pairs, and can be visualized as significant line fits with positive or negative slopes, respectively. In these scatter plots, the slope of the regression line estimates increases in the dependent variable for a unit change in the independent variable while correlation measures the strength and direction (positive or inverse) of the linear relationship, independent of the units of measurement on the two axes. Significant correlations are unrelated to slope of the regression line but rather indicate that the points in the scatter plot are closely fit to the line and provide information on whether the trend is increasing or decreasing. Panel e summarizes the data for comparisons showing statistically significant differences between correlations using repeated measures mixed regression methods with pairwise comparisons corrected with a Bonferroni correction (denoted by *, *p* < .05). No data were excluded as an outlier

Western blot data showed that NEVCs from Nulliparous KO mice (N) and from all *Loxl1* KO mice generated significantly less TIMP‐1 than NEVCs from WT mice (*p* < .0001), although no differences were noted for other assayed proteins (Figure 1). Protein‐pair ratios were not different between these groups (Figure 2).

No statistically significant correlations between individual proteins were identified within WT NEVC cells (Figure 2a). In contrast, in NEVC cultures from N mice (Figure 2b), positive correlations (i.e., line fits there were significant and had a positive slope) were noted between TIMP‐4 and MMP2 (*p* < .01), COL1A and MMP‐2, and TIMP‐1 and BMP‐1 (both *p* < .05) and negative correlations between TIMP‐4 and BMP‐1, MMP‐2 and BMP‐1 (both *p* < .01), and TIMP‐1 and TIMP‐4 (*p* < .05).

**FIGURE 3:**
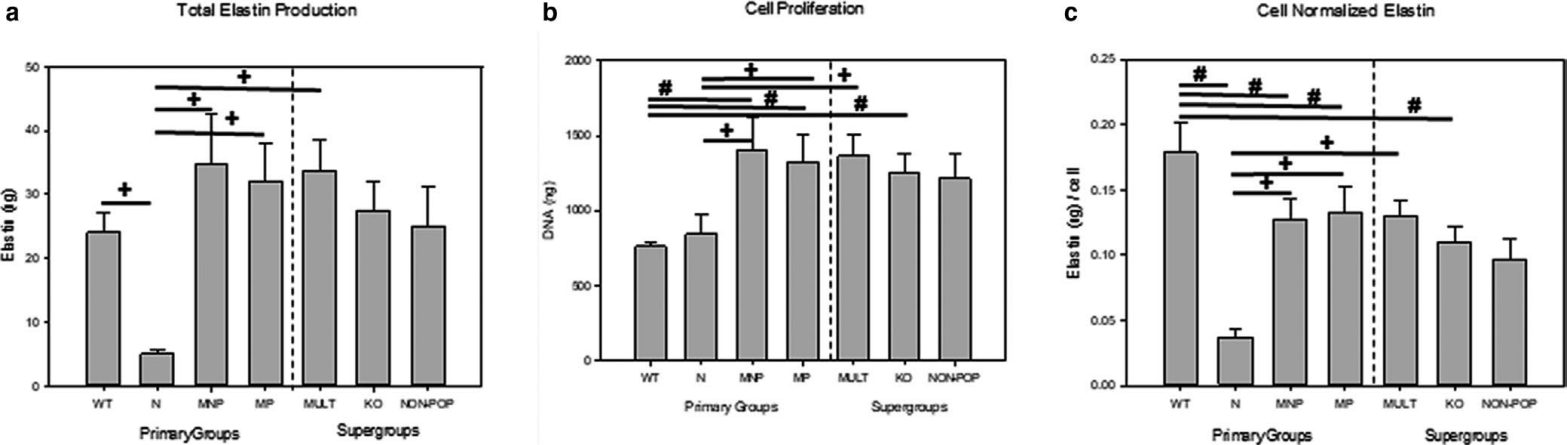
Elastic Matrix Production shown as the results of Fastin assay for total elastin (a), results of Hoechst Dye Assay for DNA Content, representing cell proliferation (b), and elastin content per cell (c) for wildtype (WT), nulliparous (N), multiparous nonprolapsed (MNP) and multiparous prolapsed (MP) mice. Bars represent mean +/− standard error of the mean of 3–6 samples/group. Repeated measures mixed regression methods with pairwise comparisons corrected using a Bonferroni correction. The following symbols denote a significant difference (*p* < .05) compared to the group in parentheses: ^#^ (wildtype; WT) and +(nulliparous; N)

Elastic matrix production normalized to cell count was significantly reduced in KO NEVC cultures versus WT NEVCs (*p* < .001) since cell proliferation was significantly increased, although total elastin production was not different between KO and WT NEVCs (Figure 3c). N NEVCs produced significantly less absolute amounts of elastic matrix versus WT NEVCs (*p* < .001; Figure 3a). Since cell proliferation was identical in both groups (Figure 3b), elastic matrix amounts synthesized on a per cell basis were also deemed significantly lower in N NEVC cultures versus WT cultures (*p* < .01; Figure 3c).

**FIGURE 4:**
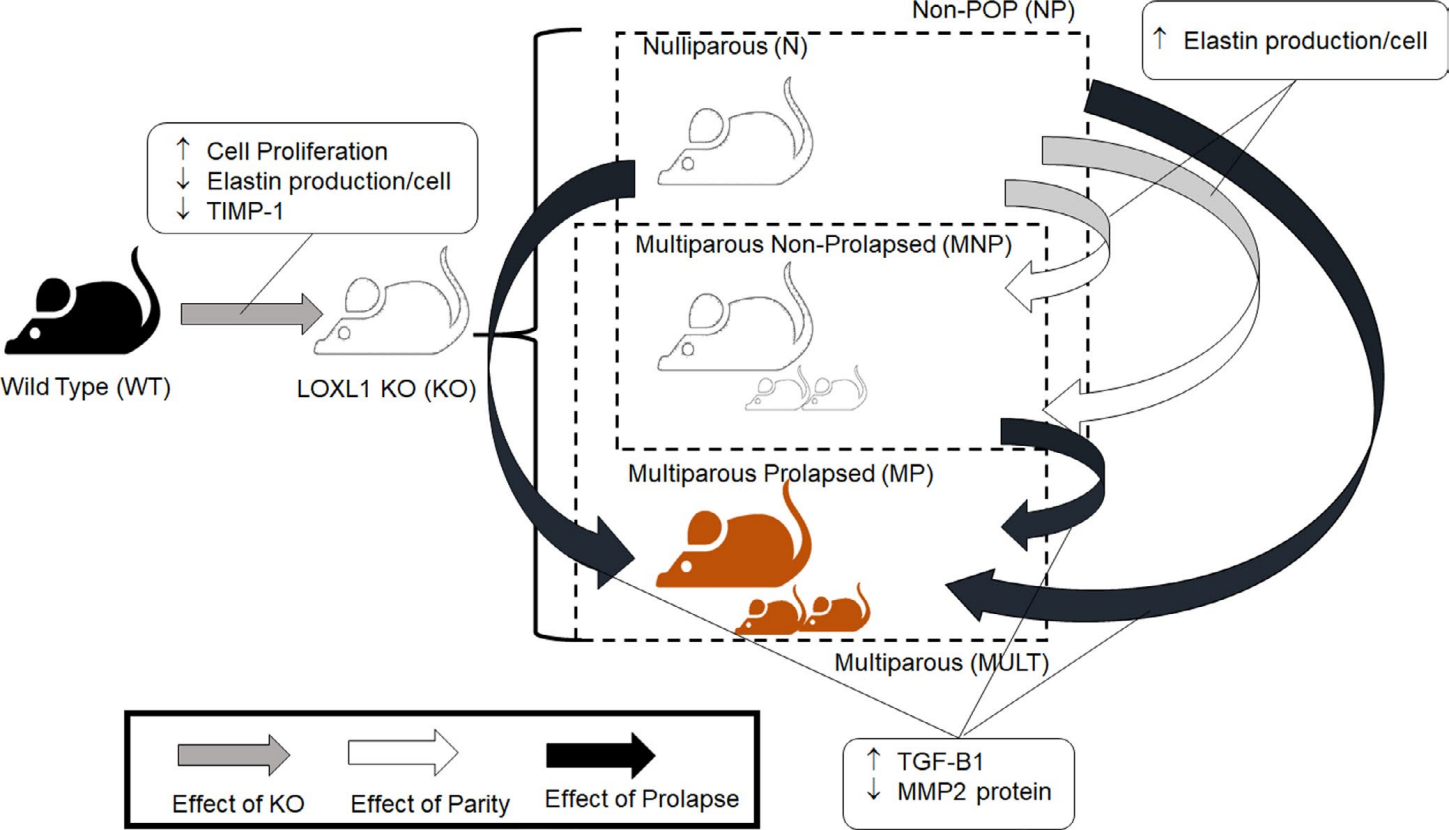
The disruption of normal elastic fiber homeostasis has been implicated in clinical manifestation of pelvic organ prolapse (POP). In this controlled study, we sought to ascertain the individual contributions of vaginal birth (parity), prolapse, and LOXL1 absence on maintenance of elastic fiber homeostasis, in a LOXL1 KO mouse model. Group‐wise comparisons relevant to each of these three effects are indicated by the respective colored arrows. We investigated changes in elastic matrix synthesis, cell proliferation, gene (RT‐PCR) and protein (western blots) expression for key elastic fiber assembly proteins, enzymes regulating matrix proteolysis and key signaling proteins (TGF‐B1 and BMP‐1) known to be implicated in elastin homeostasis. Primary outcomes pertinent to the three effects are indicated in the callouts. Our results suggest that the combination of inhibited elastin precursor synthesis, impaired cross‐linking, and poor elastin clearing creates an environment in which the vaginal ECM cannot properly repair itself after each delivery. Successive trauma sustained to the pelvic floor without proper repair could lead to POP

While our primary outcomes pertain to differences in cumulative ECM protein amounts generated by the NEVC groups over 21 days of culture (summarized in Figure 4), we also performed supplemental PCR analysis at a single, interim culture time point to assess possible congruence between genes and protein expression trends which could provide mechanistic insights. Relative to WT NEVCs, KO NEVCs showed increased expression of *Mmp2* and *Fbln5* (both *p* < .0001), as well as *Timp3, Lox,* and *Tgfb1* (all *p* < .01), and *Mmp9* and *Timp4* (both *p* < .05). N NEVCs also exhibited increased expression of the following genes compared to WT NEVCs: *Lox* and *Fbln5* (*p* < .01), as well as *Timp3*, *Timp4*, and *Fbn1* (all *p* < .05). No differences in *Eln* or *Col1a* expression were noted (Table 2).

**TABLE 2:** qRT‐PCR means comparisons, mean ± *SEM*

	*Eln*	*Col1a*	*Mmp2*	*Mmp9*	*Timp3*	*Timp4*	*Fbln5*	*Fbn1*	*Lox*	*Tgfb1*	*Bmp1*
WT	0.72 ± 2.34	1.24 ± 0.23	0.45 ± 0.31^f^	0.35 ± 1.38^f^	2.13 ± 0.71^f^	0.15 ± 0.21^f^	0.18 ± 0.11^f^	1.47 ± 0.22	0.25 ± 0.16^f^	10.8 ± 4.51^f^	1.22 ± 0.19
*N*	0.81 ± 3.14	1.53 ± 0.32	1.70 ± 0.44	1.51 ± 1.92	5.74 ± 1.00	1.13 ± 0.29	0.96 ± 0.16	2.52 ± 0.31^e^	1.25 ± 0.23	24.7 ± 6.27	1.49 ± 0.26
MP	13.3 ± 2.49^g^	1.94 ± 0.26	1.12 ± 0.35^d^, ^g^	2.26 ± 1.52^d^	6.63 ± 0.79	0.61 ± 0.23	0.92 ± 0.12	0.89 ± 0.24^g^	0.85 ± 0.18	19.1 ± 4.96^d^	1.20 ± 0.21^d^, ^g^
MNP	5.83 ± 2.09	1.92 ± 0.22	3.08 ± 0.30^c^	9.02 ± 1.30^c^	3.88 ± 0.67	0.95 ± 0.19	0.58 ± 0.10	1.59 ± 0.21	0.91 ± 0.15	42.8 ± 4.77^c^	2.51b ± 0.20^c^
MULT	8.90 ± 1.60	1.92 ± 0.17	2.26 ± 0.23	6.17 ± 0.99	5.02 ± 0.51	0.81 ± 0.15	0.72 ± 0.08	1.30 ± 0.16^b^	0.89 ± 0.12	31.4 ± 3.44	1.88 ± 0.15
KO	7.24 ± 1.43	1.84 ± 0.15	2.14 ± 0.20^a^	5.20 ± 0.88^a^	5.16 ± 0.45^a^	0.87 ± 0.13^a^	0.77 ± 0.71^a^	1.55 ± 0.14	0.96 ± 0.10^a^	29.9 ± 3.01^a^	1.79 ± 0.13
NON‐POP	4.29 ± 1.74^c^	1.80 ± 0.18	2.65 ± 0.25^c^	6.67 ± 1.07	4.45 ± 0.55	1.00 ± 0.16	0.70 ± 0.09	1.87 ± 0.17^c^	1.01 ± 0.13	36.2 ± 3.79	2.12 ± 0.16^c^

^a^ Significantly different than WT mice.

^b^ Significantly different than *N* mice.

^c^ Significantly different than MP mice.

^d^ Significantly different than MNP mice

^e^ Significantly different than MULT mice.

^f^ Significantly different than KO mice.

^g^ Significantly different than NON‐POP mice.

### 3.3 Effect of parity

Western blot analysis revealed that NEVCs from MNP mice expressed significantly more MMP‐2 (*p* < .05) and less MMP‐9 (*p* < .01) than NEVCs from N mice (Figure 1). MMP9 expression was less in NEVC cultures from MULT mice than N mice (*p* < .05); whereas, BMP1 expression was significantly greater in cells from MULT mice than N mice (*p* < .05) (Figure 1). Similarly, MMP to TIMP ratios in N, MNP, and MULT cultures were not significantly different from each other (Figure S2; https://doi.org/10.6084/m9.figshare.12130302). NEVCs from both the MP and MNP groups exhibited fewer correlations (i.e., significant line fits) between protein pairs than NEVC cultures from N mice (Figure 2b–e). There were no common protein‐pair correlations between the MNP NEVCs and both N and MP NEVC cultures (Figure 2e). A positive correlation was noted between TIMP‐1 and TIMP‐4 in both MP NEVCs and in N NEVC cultures (*p* < .001). MMP‐9 and BMP‐1 were negatively correlated in the MP cultures alone (Figure 2b–e). In the MNP NEVC group, TIMP‐1 expression positively correlated with MMP‐2 (*p* < .05) and LOX positively correlated with TGF‐β (*p* < .05) but negatively with BMP‐1 (*p* < .05; Figure 2c and e).

NEVCs from both MNP and MULT mice produced significantly more elastin measured on per cell basis or total basis than did NEVCs from N mice (both *p* < .001; Figure 3a and c). In addition, proliferation of both MNP (*p* < .01) and MULT (*p* < .05) NEVCs was significantly greater than in N NEVC cultures (Figure 3b). *Fbn1* gene expression by NEVCs in MULT cultures was significantly lower than in N cultures (*p* < .01). MNP NEVCs also showed significantly increased expression of *Bmp1* (*p* < .05) and *Mmp9* (*p* < .01) than NEVCs from N mice. A summary of significant findings for gene and protein means comparisons is provided in Figure 4.

### 3.4 Effect of prolapse

Means comparisons of the Western blot data showed that TGF‐β expression in NEVCs from MP mice was significantly higher than in NON‐POP and N NEVCs (*p* < .01). MP cultures showed significantly higher expression of TIMP‐4 compared to NON‐POP NEVCs (*p* < .05) and significantly decreased expression of MMP2 compared to both MNP (*p* < .001) and Non‐POP (*p* < .01; Figure 1). Expression ratios of all MMP and TIMP combinations, however, were not statistically different between MP, MNP, and NON‐POP cultures (Figure S2; https://doi.org/10.6084/m9.figshare.12130302). A positive correlation was noted between TIMP‐1 and TIMP‐4 in MP NEVCs, whereas N NEVC cultures exhibited a negative correlation (*p* < .001). MMP‐9 and BMP‐1 were negatively correlated in the MP cultures alone (Figure 2b, d and f).

Total and cell normalized matrix elastin production levels were not significantly different between MP, MNP, and NON‐POP cultures (Figure 3a and c). MP NEVCs produced significantly more elastic matrix both on a total and per cell basis than N NEVCs (both *p* < .001), and showed significantly greater proliferation (*p* < .05; Figure 3a–c).

NEVCs from MP mice showed significantly elevated *Eln* gene expression (*p* < .05) and decreased expression for *Mmp2, Fbn1*, and *Bmp1* (*p* < .01), as well as *Tgfβ1* (*p* < .05) compared to NEVCs from NON‐POP mice (Table 2). Similarly, MP cells also expressed significantly more *Eln* (*p* < .01) and less *Fbn1* (*p* < .001) than N cells. NEVCs from MP mice also exhibited significantly decreased gene expression for *Mmp2* and *Bmp1* (*p* < .001), as well as *Mmp9* and *Tgfβ1* (*p* < .01) compared to those from MNP mice. A summary of significant findings for gene and protein means comparisons is available in Figure 4.

## 4 DISCUSSION

Despite the high prevalence rates of POP, its pathophysiology is not well understood. It is well documented that risk factors such as vaginal parity, age, and family history are the largest predictors of POP (Hallock & Handa, 2016), however it is currently unknown how the pathophysiology of the disease relates to them. With the long‐term goal of elucidating the biochemical pathophysiology of POP, this study focused on identifying key changes in elastic fiber metabolism in mouse vaginal tissue as they related to the absence of the *Loxl1* gene, the influence of multiparity, and the presence of vaginal prolapse.

To isolate the effect of the absence of LOXL1, expression of key proteins involved in elastin homeostasis by WT and N cells was directly compared. When lacking LOXL1, NEVCs reliably generated significantly less elastin on a total and per cell basis compared to WT NEVCs. Additionally, in the absence of both LOXL1 and any contributions from parity, the MMP‐9/TIMP‐1 ratio was significantly higher, suggesting a mechanistic relationship between LOXL1 and these proteins. A tightly controlled relationship between MMP‐9 and TIMP‐1 has already been documented (Wieslander et al., 2008), with high MMP‐9/TIMP‐1 ratios associated with impaired fibrosis in lung tissue (Corbel et al., 2000), slower wound healing in skin (Yang et al., 2009), and a weakening of fetal membrane tensile strength during pregnancy (Institute of Medicine (US) Committee, 2007). Not surprisingly, it has been shown that in addition to developing POP, *Loxl11* KO mice typically exhibit much larger lung airspaces and more compliant skin tissue (Liu et al., 2004), highlighting LOXL1’s role in maintaining tissue homeostasis beyond the vaginal wall.

It is possible that these proteins are linked via their interaction to TGF‐β which has been shown to regulate LOXL1, LOX, and TIMP‐1 during ECM regeneration (Choudhary et al., 2009; Goto et al., 2005; Ramamurthi et al., 2012; Shanley et al., 1997; Zenkel et al., 2011) and is also activated by MMP‐9 (Page‐McCaw et al., 2007; Parks et al., 2004). In normal ECM regeneration, latent TGF‐β that has been sequestered in the fibrillin scaffold of mature elastic fibers (Dijke & Arthur, 2007) is released and activated, resulting in increases in LOX activity (Shanley et al., 1997) and TIMP‐1 production (Xue & Jackson, 2015) while variably affecting MMPs (Krstic & Santibanez, 2014; Thompson et al., 2000). LOX and LOXL1 in turn may enhance the function of latent TGF‐β binding protein (LTBP), resulting in more TGF‐β activation and ECM development (Atsawasuwan et al., 2008; Zenkel et al., 2011). It is possible that the absence of LOXL1 in KO mice, combined with no compensatory increases of LOX protein, could lead to reduced TGF‐β activation, explaining the reduction in TIMP‐1 expression.

Interestingly, many of the effects seen with the deletion of the *Loxl1* gene were reversed in multiparous KO mice. The amount of total elastin produced by cells cultured from MNP and MP mice was not significantly different from those of WT mice. This was in part due to significant increases in cell proliferation that occurred in both multiparous groups, as cell normalized elastin production remained below WT levels despite being significantly increased with respect to N cells. This parity‐induced increase in cell proliferation, which occurs despite the relatively short time scale of mouse gestation and postpartum recovery, has not been previously reported.

Because LOXL1 is the primary protein involved in postpartum tropoelastin cross‐linking, whereas LOX is more important for prenatal development (Behmoaras et al., 2008; Liu et al., 2004), *Loxl1* KO mice are unable to organize the excess tropoelastin molecules produced as a result of delivery into mature fibers (Drewes et al., 2007). The continual presence of tropoelastin aggregates has been shown to promote SMC activation into a proliferative state (Hinek et al., 2000; Mochizuki et al., 2002), while the presence of insoluble elastin restores the quiescent phenotype (Hinek et al., 2000; Karnik et al., 2003). We hypothesize that vaginal SMCs from *Loxl1* KO mice, unable to organize tropoelastin to mature cross linked elastin, are permanently fixed in a proliferative phenotype, leading to the increased proliferation we observed in NEVCs from MNP and MP mice.

While new methods (e.g., Ki‐67 measurement) are now available to estimate cell proliferation (Adan et al., 2016), these are useful to indicate the proliferation “state” of cells at any point of time. Nonetheless, the Hoechst dye‐based DNA Assay developed by Labarca and Paigen (1980) remains relevant. Although it is not useful for assessing dynamic changes in cell proliferation states of the different groups of cells, it enables measurement of differences in cumulative cell proliferation over a defined period of culture on the same harvested cell layer sample as matrix assays are performed. Therefore, this DNA assay allowed us to normalize measured matrix amounts to allow for reliable comparison between cell culture groups.

No differences in any MMP/TIMP ratios were observed between the multiparous and the N mouse groups, suggesting a limited effect of parity on these relationships. TIMP‐1 expression remained decreased with parity. MMP‐9, however, returned to WT levels while MMP‐2 levels rose, highlighting the effect of childbirth on MMP regulation as a crucial aspect of the postpartum remodeling process. During delivery, damage to the elastic matrix results in the accumulation of small fragmented elastin like polypeptides (ELPs) (Drewes et al., 2007). These ELPs act as chemo attractants for both fibroblasts and MMP‐expressing immune cells such as monocytes and macrophages to initiate the remodeling process (Drewes et al., 2007; Duca et al., 2004; Hance et al., 2002). Furthermore, Rahn *et al*. have demonstrated that vaginal distension, such as during parturition, upregulates MMP‐2 and MMP‐9 expression (Papke & Yanagisawa, 2014) which corroborates both our data and that available in literature (Dubicke et al., 2008; Ekman‐Ordeberg & Dubicke, 2012; Wieslander et al., 2008).

Finally, we compared the protein expression of NEVCs obtained from MNP mice to MP mice to determine differences in relation to development of vaginal prolapse. No differences in either total or cell normalized elastin production as well as in cell proliferation were observed between the two groups. Surprisingly, the only protein that was differentially expressed was MMP‐2, which was significantly decreased in prolapsed mice. While this is contrary to both mouse and human studies that have found an increase in MMP production in prolapsed tissues (Atsawasuwan et al., 2008; Budatha et al., 2011; Chen et al., 2002; Wagenseil & Mecham, 2007), it is to be noted that much of the available literature reports variable results (Chen & Yeh, 2011).

Additionally, NEVCs from MP mice expressed significantly more TGF‐β than those from N mice. MNP cells, however, exhibited no such surge of expression. In contrast, MNP cells expressed significantly less MMP‐9 than N cells; whereas no similar relationship was seen between MP and N cells. These findings show a clear distinction in how MP and MNP cells respond to parturition, suggesting that the development of POP results from an aberrant biochemical response to the mechanical insult associated with vaginal childbirth.

The nonpathogenic postpartum remodeling process consists of a complex coordination of structural and catalytic proteins that must remain in delicate balance. Paramount to the successful creation of new elastic matrix are the effects of MMPs and TIMPs on vaginal and pelvic floor ECM. In healthy wild type mice, *Mmp2* gene expression has been shown to be upregulated 12–24 hr after delivery, while *Mmp9* gene expression surges roughly 24 hr later (Wieslander et al., 2008), comprising two distinct waves of ECM breakdown. Additionally, experiments in rats have shown that myometrial *Timp4* gene expression is significantly upregulated 1 day after delivery, contrasting with a decrease in *Timp1* gene expression (Nguyen et al., 2016). These findings coupled with the specific inhibitory abilities of TIMP‐4 on proMMP‐2 and TIMP‐1 on proMMP‐9 (Brew & Nagase, 2010) describe a model of postpartum involution that helps explain the pathological nature of our own findings in *Loxl1* KO mice.

Within the first day after delivery in normal mice, *Mmp2* gene expression increases, followed by an upregulation of *Timp4* after 24 hr to control the initial degradation (Nguyen et al., 2016; Wieslander et al., 2008). A second wave is then initiated by *Mmp9* upregulation 1–2 days after delivery, facilitated by a drop in *Timp1* expression (Nguyen et al., 2016; Wieslander et al., 2008). The reduced MMP‐2 expression in our cultures of MP cells could possibly suggest that these mice failed to initiate the first step of the remodeling process. Increased TGF‐β in these cultures could possibly detrimentally affect the MMP feedback mechanism by suppressing MMP activity and increasing TIMP‐4 activity (Huang et al., 2011; Rudolph‐Owen et al., 1997).

In this study we have proposed a model that links biochemical aberrations in NEVCs to biomechanical disorders of the pelvic floor in mice. The application of the *Loxl1* KO mouse model to human tissues, however, is limited. While recent research has shown that a decrease in LOXL1 accompanies POP development in women (Jung et al., 2009; Klutke et al., 2008; Kow et al., 2016; Kufaishi et al., 2016; Zhao & Zhou, 2012) a number of studies in humans have produced results different from our own with regards to expression of proteins such as LOX (Kobak et al., 2005) and MMPs (Atsawasuwan et al., 2008; Budatha et al., 2011; Chen et al., 2002). Thus, while *Loxl1* KO mice remain a useful and relevant model of human prolapse, they do not exactly replicate the conditions leading to prolapse in women.

Additional limitations of the study stem from the experimental design itself. The small number of animals used in each group and overall variability of the results reduced overall precision of measurements, potentially resulting in reduced accuracy and possibly some missed significant results. Since we did not find any experimental reason to justify removal of any of the records, all the data were used for the analysis and outliers were not removed. The small sample volumes to test expression of a large number of genes, that too in replicate, also limited us to using a single housekeeping gene for PCR. In this study, we have selected a housekeeping gene, 18s, for normalizing target gene expression, due to its relative stability of expression versus other housekeeping genes such as GAPDH (Barber et al., 2005) and because, it is stably expressed across our tested NEVC lines (*p* = .106). However, our future studies will test additional housekeeping genes to further reduce normalization error towards enhancing the value of our gene expression data.

The small sample size in this study, however highlights the significance of the differences that were present. Furthermore, a WT multiparous group for comparison of the normal stages of postpartum involution to those of prolapsed mice was absent and could be included in future studies. We used WT mice on a comparable genetic background but not from the same breeding colony, which may have added variability to the study. We have since moved to use heterozygote breeding to obtain genetically matched WT and KO mice. Additional future directions could include a quantitative histological analysis of elastic fiber matrix with regard to elastic matrix structural integrity in mice and/or humans with and without POP to test the hypothesis of major ECM disarray as the main contributor to POP. Additional studies could include artificial upregulation of TIMPs or knock down of MMPs at the beginning of the involution stages of WT multiparous mice to potentially recreate the symptoms seen in MP mice.

In summary, we hypothesize that the failure of NEVCs to produce sufficient amounts of MMP‐2 to initiate the postpartum remodeling process results in the accumulation of damaged elastin fibers that serve as an improper scaffold for future tropoelastin deposition. It has been shown that prolapsed mice exhibit fragmented and disoriented elastin fibers as early as 2 hr after delivery (Drewes et al., 2007) and for as long as one week postpartum (Liu et al., 2004). In normal mice, the early postpartum period is characterized by a burst of tropoelastin production and elastin fiber cross‐linking, occurring as early as 24 and 48 hr after delivery, respectively (Drewes et al., 2007) roughly the same temporal window as the MMP‐2 peak (Wieslander et al., 2008).

We have shown that multiparous *Loxl1* KO mice produce reduced levels of elastin compared to WT mice on a per cell basis. The combination of inhibited elastin precursor synthesis, impaired cross‐linking, and poor elastin clearing creates an environment in which the vaginal ECM cannot properly repair itself after each delivery. Successive trauma sustained to the pelvic floor without proper repair could lead to POP.

## CONFLICT OF INTEREST

The authors have no conflicts of interest to declare relative to this work.

## AUTHOR CONTRIBUTIONS

S.A.J., G.S., S.B., M.S.D., and A.R. analyzed the data. S.A.J, G.S., and S.B. drafted the manuscript. M.S.D. and A.R. edited and revised the manuscript. S.A.J., G.S., S.B., M.S.D., and A.R. approved the final version. G.S., M.S.D., and A.R. conceived and designed the research. G.S., S.D., B.C., and M.K. conducted the experiments.

## Supporting information



Figure S1‐S2
