# Supplementary material for: Elastin homeostasis is altered with pelvic organ prolapse in cultures of vaginal cells from a lysyl oxidase‐like 1 knockout mouse model
Source: Physiol Rep. 2020 Jun 12;8(11):e14436. doi: 10.14814/phy2.14436 (PMC7292929; doi:10.14814/phy2.14436)
Supplement: Supplementary file 1 — Figure S1‐S2 [file PHY2-8-e14436-s001.docx]

**Figure S1**. (<https://doi.org/10.6084/m9.figshare.11798871>) Immunofluorescence images showing with that NEVCs express Myosin Heavy Chain 11 (mature SMC marker), and less so, Fibroblast-Specific Protein-1 (FSP-1), but not pan-cytokeratin antibodies (AE-1/ AE-3) that are expressed only by epithelial cells. These results demonstrate that our vaginal cells are composed of SMCs and fibroblasts for which reason they are termed as nonepithelial vaginal cells (NEVCs).

**Figure S2.** (<https://doi.org/10.6084/m9.figshare.12130302>) **MMP/TIMP Protein Ratios** of MMP2/TIMP1 (**A**), MMP2/TIMP4 (**B**), MMP9/TIMP1 (**C**), MMP9/TIMP4 (**D**). Each bar in the box plot represents mean with 25/75% confidence interval, blue dotted lines indicating mean. Data were collected from 3–5 nonepithelial vaginal cells (NEVCs) in each group. Each cell line has three replications. NEVCs from wildtype (WT) mice are indicated as triangles to distinguish them from NEVCs in the other groups (squares) all of which are from lysyl oxidase-like 1 knockout mice. The same color in the symbols corresponds to samples from the same cell line. Repeated measures mixed regression methods with pairwise comparisons corrected using a Bonferroni correction. There were no significant differences between the groups in MMP/TIMP protein ratios.
